# On the Varieties of Headache

**Published:** 1847-09

**Authors:** 


### ON THE VARIETIES OF HEADACHE.

An interesting article on this subject will be found in another department of this number. Dr. Wright has described with great clearness several varieties of headache. We have often met with the following not mentioned by him: An individual rises in the morning with a headache, with which he may or may not have gone to bed. At dinner or supper the evening before he partook of some indigestible article of food which, passing the stomach, is producing irritation in the bowels. In this region green corn is often the cause of such trouble. The remedy is obvious, and consists in a laxative to remove the offending body. A Seidlitz powder or a little Bluelick water, taken before breakfast operates promptly and affords complete relief.

We have met with still another variety of headache, which generally attended influenza in 1843, and is often associated with catarrh. This is a headache confined to a small space over the eye, in the region of the frontal sinuses, and is not unfrequently periodical in its character. Such headache is sometimes purely nervous, and will yield to an opiate. In the epidemic referred to, this pain in the forehead was the most distressing symptom in a majority of cases, and its subsidence under a teaspoonful or two of paregoric often surprised as much as it delighted the patient. It was found necessary to repeat the opiate two or three days at the recurrence of the headache.

